# Nitric oxide modulating ion balance in *Hylotelephium erythrostictum* roots subjected to NaCl stress based on the analysis of transcriptome, fluorescence, and ion fluxes

**DOI:** 10.1038/s41598-019-54611-2

**Published:** 2019-12-04

**Authors:** Zhixin Chen, Xueqi Zhao, Zenghui Hu, Pingsheng Leng

**Affiliations:** 1 Beijing Advanced Innovation Center for Tree Breeding by Molecular Design, Beijing University of Agriculture, Beijing, 102206 China; 2 College of Landscape Architecture, Beijing University of Agriculture, Beijing, 102206 China; 3 Beijing Collaborative Innovation Center for Eco-environmental Improvement with Forestry and Fruit Trees, Beijing, 102206 China; 4 Beijing Laboratory of Urban and Rural Ecological Environment, Beijing, 102206 China

**Keywords:** Plant physiology, Plant stress responses

## Abstract

Soil salinization is one of the main stress factors that affect both growth and development of plants. *Hylotelephium erythrostictum* exhibits strong resistance to salt, but the underlying genetic mechanisms remain unclear. In this study, hydroponically cultured seedlings of *H. erythrostictum* were exposed to 200 mM NaCl. RNA-Seq was used to determine root transcriptomes at 0, 5, and 10 days, and potential candidate genes with differential expression were analyzed. Transcriptome sequencing generated 89.413 Gb of raw data, which were assembled into 111,341 unigenes, 82,081 of which were annotated. Differentially expressed genes associated to Na^+^ and K^+^ transport, Ca^2+^ channel, calcium binding protein, and nitric oxide (NO) biosynthesis had high expression levels in response to salt stress. An increased fluorescence intensity of NO indicated that it played an important role in the regulation of the cytosolic K^+^/Na^+^ balance in response to salt stress. Exogenous NO donor and NO biosynthesis inhibitors significantly increased and decreased the Na^+^ efflux, respectively, thus causing the opposite effect for K^+^ efflux. Moreover, under salt stress, exogenous NO donors and NO biosynthesis inhibitors enhanced and reduced Ca^2+^ influx, respectively. Combined with Ca^2+^ reagent regulation of Na^+^ and K^+^ fluxes, this study identifies how NaCl-induced NO may function as a signaling messenger that modulates the K^+^/Na^+^ balance in the cytoplasm via the Ca^2+^ signaling pathway. This enhances the salt resistance in *H. erythrostictum* roots.

## Introduction

Soil salinization is one of the main environmental stressors for plants, and one of the main factors that restrict agricultural and forestry production. Currently, about 7% of the total world land area is threatened by salinization, and a figure has been predicted to increase at a rate of 1–1.5 million hm^2^ per year. China is particularly affected by soil salinization, and approximately 7.6 million hm^2^ of farmland (almost 1/5 of the total) shows certain degrees of salinization. Salt stress severely reduces plant growth and survival by causing osmotic stress and ion toxicity^1^. Investigating the cellular and genetic mechanisms of salt-resistance is an important step in the development of new varieties with improved resistance to salt-stress.

Many physiological, biochemical, and molecular processes contribute to the salt resistance of plants^2^. Among these are the Ca^2+^ signaling pathway^3^, the salt overly sensitive (SOS) pathway^4^, and plant hormone signaling pathway^5^. The expression regulation of many specific genes associated with salt resistance have also been investigated, and a number of these were effectively used to improve salt resistance of plants^6,7^. In general, plant salt resistance is mediated by polygenic pathways, which produce salt-resistance proteins. As a popular method, transcriptome sequencing has been widely used to explore the potential mechanisms involved in the integral plant salt resistance. High-throughput transcriptome analysis has been performed in many plant species, such as *Gossypium davidsonii*^8^, *Oryza sativa*^9^, *Hordeum spontaneum*^10^, *Pyrus betulaefolia*^11^, *Petunia hybrida*^12^, and *Vitis vinifera*^13^. The main aim is to scan the transcribed genes and their expression in a global context under salt stress.

Plant root is the primary part that comes into contact with ionic toxicity and osmotic stress in response to high soil salinity. Upon perceiving salt stress, the root transduces the salt-stress signal upwards, thus rapidly initiating a defensive mechanism in the whole plant. Thus, the root response to salt stress plays an important role in the salt-resistance mechanism of plants. In the NaCl-stressed roots of *Glycyrrhiza uralensis* a multiple salt-sensitive signaling network regulates the K^+^/Na^+^ homeostasis gene expression^14^. The high-throughput sequencing of mRNA (RNA-Seq) has been used to analyze transcriptome changes of NaCl-treated roots to understand the molecular basis of the observed salt tolerance^15^. After exposure to NaCl, 6,547 genes are shown to be differentially regulated in *Avicennia officinalis* roots based on RNA-sequencing, which implies that ABA-independent signaling pathways played a key role in salt tolerance^15^. Similarly, 13,522 differentially expressed genes (DEGs) are identified in the *Iris lactea* var. *chinensis* roots after NaCl stress, including 7,037 up-regulated and 6,539 down-regulated DEGs^16^. Transcriptome analysis has been used to reveal the responding signaling pathways and biological processes to salt stress in *Arachis hypogaea* roots^17^. RNA-seq technology has also been used to analyze the gene expression map of *Citrus* roots after salt treatment^18^. SOS, reactive oxygen species (ROS), hormone signaling pathways, a multitude of transcription factors, and genes related to cell wall loosening and stiffening are found to be involved in the salt stress response^18^.

Nitric oxide (NO) is an important gaseous signal molecule, which plays a key role in a number of physiological processes in plants. These include root formation, seed germination, seedling growth, stomatal closure, maturation, flowering, senescence, programmed cell death, as well as biotic and abiotic stress responses^19–21^. It has been reported that NO contributes to the salt stress tolerance in plants^22^. NO mediates melatonin-enhanced tolerance to salinity stress in *Brassica napus* seedlings^23^. NO alleviates salt stress by regulating the levels of osmolytes and antioxidant enzymes in the chickpea^24^. Exogenous NO delays salt-induced leaf senescence in *G. hirsutum* through increases in chlorophyll content, K^+^ content, photosynthetic rate, and LHCB gene expression^25^. Most of the previous studies focus on the effects of NO on the physiochemical indexes under NaCl stress; however, its role in ion balance modulation in roots remains largely unknown.

Plants of the Crassulaceae family display a preference for stress-prone environments (i.e., low water and high salinity). The Crassulacean acid metabolism (CAM) pathway resulted in resistance, especially the strong drought-resistance^26,27^. It has also been reported that Crassulaceae plants have high salt-resistance^28^. *Hylotelephium erythrostictum* is a perennial succulent herb of the Crassulaceae family. *H. erythrostictum* not only possesses strong stress-resistance, but also has high ornamental value; therefore, it has been used widely in urban greening and ecological landscaping in China. *H. erythrostictum* has been found to grow well in the high-salinity soil of the northern cities of China, especially in the coastal areas of Tianjin and Tangshan, which suggests high salt-resistance. Su *et al*. reported that the salt tolerance threshold and limit values of *H. erythrostictum* seeds were 1.47% and 1.97%, respectively^29^. Chen *et al*. reported that hydroponic seedlings of *H. erythrostictum* grew well in a 50 mM NaCl solution^30^. However, in CAM plants, the salt-resistance mechanisms in ion balance modulation of roots, especially the role of NO remains little understood. In this study, after treatment with 200 mM NaCl for 0 (T0, control), 5 (T5), and 10 (T10) days, RNA-seq was performed to identify the potential candidate genes related to ion transportation and NO biosynthesis that have differential expressions in the roots of hydroponic *H. erythrostictum* seedlings. Furthermore, combined with NO reagents, the fluorescence of NO and ion fluxes were tested to determine the role of NO in ion balance modulation. This study provides important information toward discovering the salt-resistance mechanisms in the roots of CAM plants. This information is used to explore the pathways for enhancing the salt-resistance of other horticultural plants to utilize and improve high-salinity soil.

## Results

Based on transcriptome analysis, a total of 89.413 Gb of raw bases and 596.092 Mb of raw reads were obtained, averaging at 9.935 Gb and 66.232 Mb, respectively (Supplementary Table S1). After discarding low-quality reads (containing adapters and unknown or low-quality bases) and after stringent quality checks and data cleaning, a total of 81.523 Gb clean bases and 571.719 Mb of clean reads were obtained (Supplementary Table S1). Based on the high quality reads, using paired-end joining and gap-filling, 177,053 transcripts and 111,341 unigenes were assembled with different length distribution (Supplementary Table S2). Then, all unigenes were aligned to seven protein databases containing Nr, TrEMBL, Swiss-Prot, Pfam, KOG, GO, and KO using BLAST with an E-value threshold of 10^−5^, and 82,081 unigenes were annotated  (Supplementary Fig. S1). The functions of unigenes were classified by Gene Ontology (GO, Supplementary Fig. S2). Based on the homology, the unigenes were categorized into 56 groups. The KEGG database presented the pathways of molecular interactions and reactions, which (based on the comparison of unigenes) were assigned to different pathways. These were classified into six categories, including Organismal Systems, Metabolism, Human Diseases, Genetic Information Processing, Environmental Information Processing, and Cellular Processes (Supplementary Fig. S3). DEGs between different treatment times were compared and analyzed. The volcano plots of DEGs between samples are shown in Supplementary Fig. S4A, and the DEGs were also identified between two samples by comparing T0 and T5, T5 and T10, and T0 and T10 (Supplementary Fig. S4B). Based on the enrichment of DEGs, the up-regulated GO terms and KEGG pathways outnumbered those that were down-regulated between T0 and T5, T5 and T10, and T0 and T10 (Supplementary Table S3).

**Figure 1:**
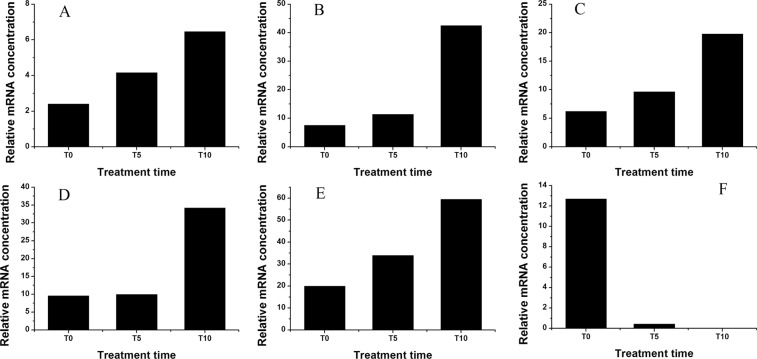
Expression profiles of DEGs annotated to Na^+^ and K^+^ transporters based on RNA-Seq. (**A**), TR9124|c0_g1 (*SOS1*); (**B**), TR19254|c2_g1 (*AKT1*); (**C**), TR11778|c0_g1 (*NHX1*); (**D**), TR6184|c2_g1 (*NHX2*); (**E**), TR13899|c2_g3 (*NHX3*); (**F**), TR8982|c1_g1 (*HKT1*). T0, T5, and T10 indicates that the samples are treated by 200 mM NaCl for 0, 5, and 10 days.

Unigenes related to K^+^ and Na^+^ transporters were identified among the DEGs (Fig. 1). The expression level of TR9124|c0_g1, annotated to a plasma membrane-localized Na^+^/H^+^ antiporter salt overly sensitive 1 (*SOS1*), exhibited a gradual increasing pattern with treatment time. The relative mRNA concentrations of *SOS1* at T5 and T10 increased by 72.81% and 167.98%, respectively, compared with that at T0. Moreover, expressions of the unigenes annotated to the tonoplast-localized Na^+^/H^+^ exchanger (*NHX*), such as TR11778|c0_g1 (*NHX1*), TR6184|c2_g1 (*NHX2*), and TR13899|c2_g3 (*NHX3*), were also enhanced after salt stress. The relative mRNA concentrations of TR11778|c0_g1, TR6184|c2_g1, and TR13899|c2_g3 at T10 were about 3.17, 3.58, and 2.97-fold of those at T0, respectively. The unigene TR19254|c2_g1, annotated to the membrane-localized inward-rectifying K^+^ channel (*AKT1*), was up-regulated by the salt treatment, and its relative mRNA concentration at T10 was 4.60-fold higher than at T0. However, the unigene TR8982|c1_g1, annotated to the high-affinity K^+^ transporter (*HKT1*), was down-regulated after salt treatment, and its relative mRNA concentration decreased from 12.69 at T0 to 0 at T10.

**Figure 2:**
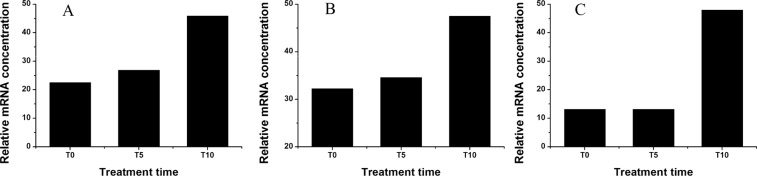
Expression profiles of DEGs annotated to the Ca^2+^ channel based on RNA-Seq. (**A**), TR5140|c0_g1 (Ca^2+^ channel-1); (**B**), TR5837|c0_g1 (Ca^2+^ channel-2); (**C**), TR8424|c0_g3 (Ca^2+^ channel-3).

Three unigenes were found to be annotated to the Ca^2+^ channel (TR5140|c0_g1, TR5837|c0_g1, and TR8424|c0_g3) and also presented high expression levels after NaCl treatment (Fig. 2). The relative mRNA concentrations of TR5140|c0_g1, TR5837|c0_g1, and TR8424|c0_g3 at T10 were about 1.03, 0.47, and 2.6-fold higher than at T0.

**Figure 3:**
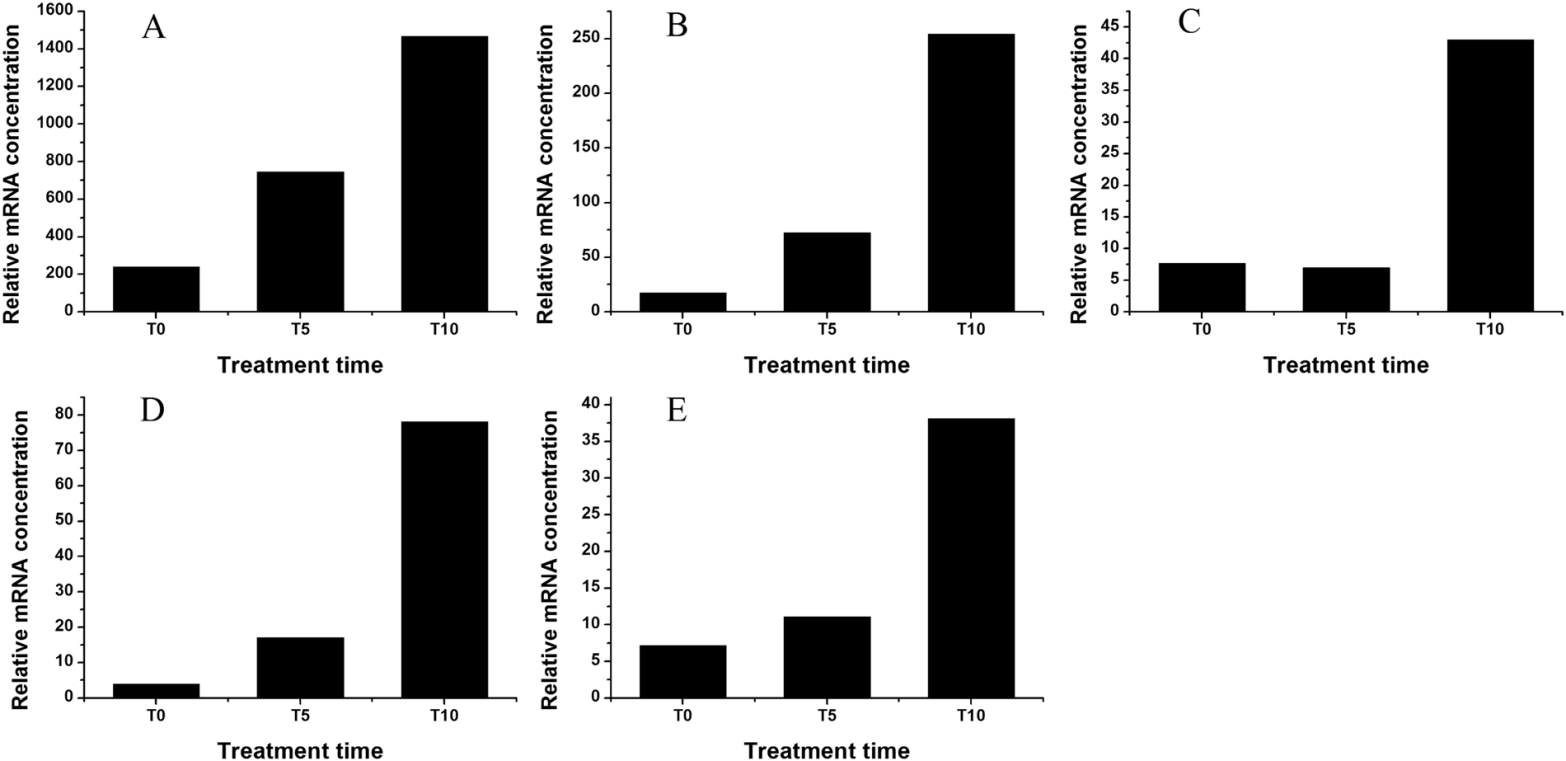
Expression profiles of DEGs annotated to the calcium binding protein based on RNA-Seq. (**A**), TR5531|c0_g5 (calmodulin, *CAM*); (**B**), TR27565|c0_g2 (calmodulin-like protein, *CML*); (**C**), TR11824|c1_g1 (calcineurin B-like protein, *CBL*); (**D**), TR27162|c2_g4 (CBL-interacting protein kinase, *CIPK*); (**E**), TR12612|c0_g4 (calcium-dependent protein kinase, *CDPK*).

The unigenes, annotated to calcium binding proteins in the calcium signaling pathway, were also triggered by NaCl treatment (Fig. 3). After exposure to NaCl, TR5531|c0_g5, annotated to calmodulin (*CAM*), TR27565|c0_g2 annotated to calmodulin-like protein (*CML*), TR11824|c1_g1 annotated to calcineurin B-like protein (*CBL*), TR27162|c2_g4 annotated to CBL-interacting protein kinase (*CIPK*), and TR12612|c0_g4 annotated to calcium-dependent protein kinase (*CDPK*) had high relative mRNA concentrations. With the exception of TR11824|c1_g1, the expression levels of the other four unigenes gradually increased along with treatment time. The highest expressions of these five unigenes all occurred at T10. The expression levels of TR5531|c0_g5, TR27565|c0_g2, TR11824|c1_g1, TR27162|c2_g4, and TR12612|c0_g4 at T10 were about 6.09, 14.50, 5.56, 19.54, and 5.33-fold of those at T0, respectively.

**Figure 4:**
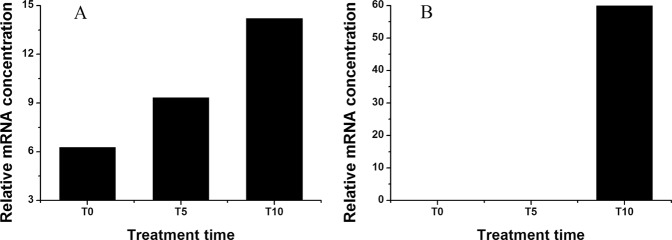
Expression profiles of DEGs involved in NO biosynthesis based on RNA-Seq. (**A**), TR10047|c0_g1 (*NOA1*); (**B**), TR23909|c0_g1 (*nitrate reductase*, *NR*).

The expression levels of unigenes involved in the NO biosynthesis increased after NaCl treatment (Fig. 4). The relative mRNA concentration of TR10047|c0_g1, which was annotated to nitric oxide associated (*NOA1*), increased by 50.0% and 127.0% at T5 and T10, respectively. At T0, TR23909|c0_g1 (nitrate reductase, *NR*) was not expressed, while its relative mRNA concentration exceeded 60 at T10.

**Figure 5:**
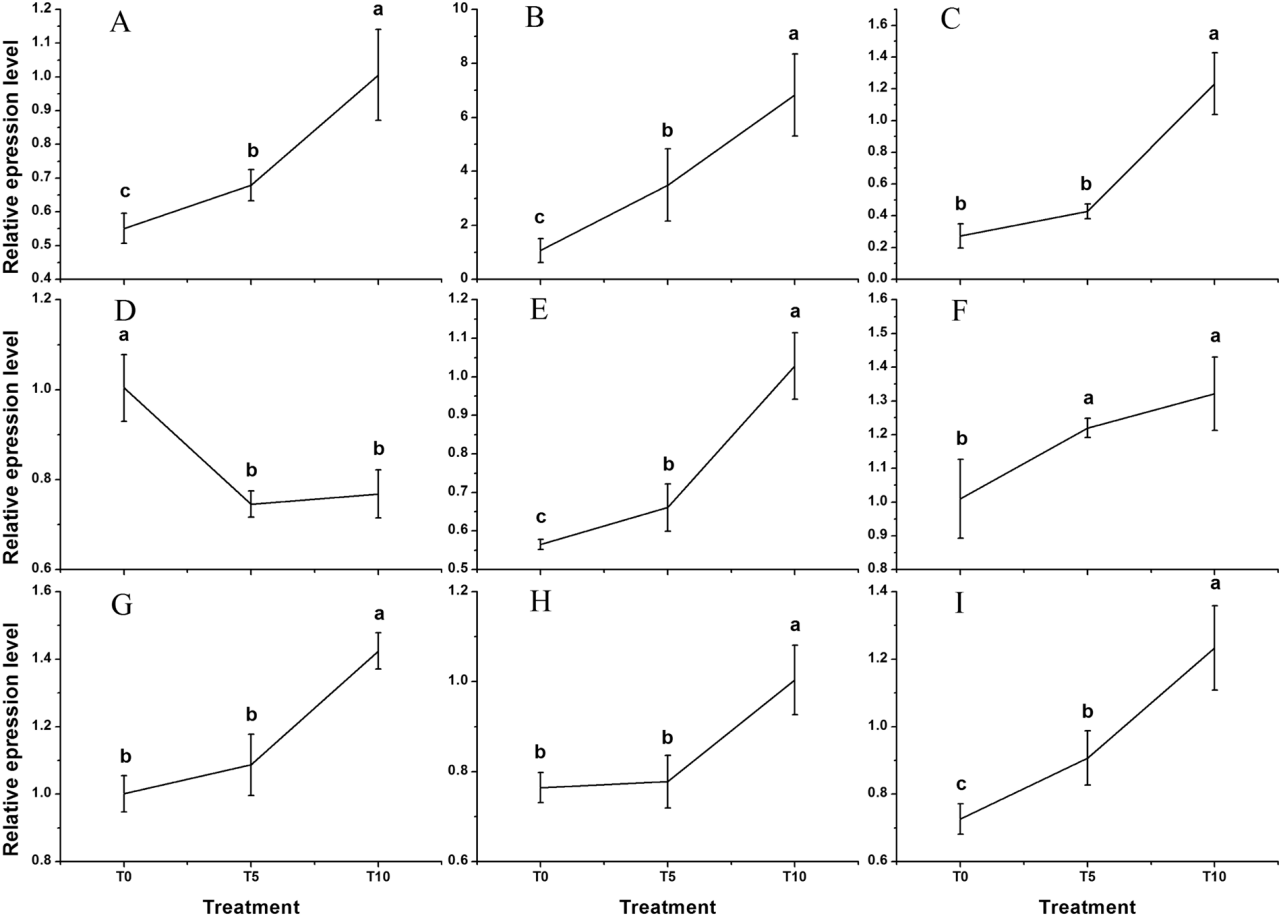
Relative expression levels of nine DEGs based on qRT-PCR. (**A**), TR9124|c0_g1 (*SOS1*); (**B**), TR19254|c2_g1 (*AKT1*); (**C**), TR11778|c0_g1 (*NHX1*); (**D**), TR8982|c1_g1 (*HKT1*); (**E**), TR5140|c0_g1 (Ca^2+^ channel); (**F**), TR5531|c0_g5 (*CAM*); (**G**), TR11824|c1_g1 (*CBL*); (**H**), TR12612|c0_g4 (*CDPK*); (**I**), TR10047|c0_g1 (*NOA1*). The bars represent the means of three independent biological replicates, and the error bars represent standard errors. Statistical significance [least significant difference (LSD)] of the difference in relative expression levels under different treatments is indicated by different small letters (*P* < 0.05).

Nine DEGs were selected to validate the expression patterns that were obtained by RNA-Seq based on qRT-PCR analysis. The expression profiles of TR9124|c0_g1, TR19254|c2_g1, TR11778|c0_g1, TR8982|c1_g1, TR5140|c0_g1, TR5531|c0_g5, TR11824|c1_g1, TR12612|c0_g4, and TR10047|c0_g1 of *H. erythrostictum* roots at different treatment times are shown in Fig. 5. The expression patterns of these genes were similar to the results of RNA-seq.

**Figure 6:**
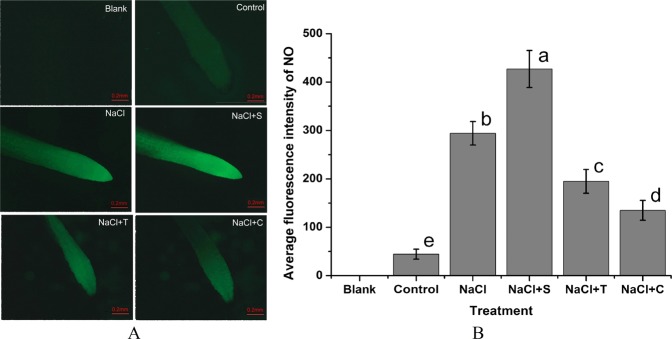
Effect of NaCl and NO reagents on the NO fluorescence (**A**) and the average fluorescence intensity (**B**) in root tips of *H. erythrostictum*. Blank, no DAF-FM DA and NaCl; Control, adding DAF-FM DA without NaCl treatment; NaCl, adding DAF-FM DA after NaCl treatment; NaCl + S, adding DAF-FM DA after NaCl + SNP treatment; NaCl + T, adding DAF-FM DA after NaCl + sodium tungstate treatment; NaCl + C, adding DAF-FM DA after NaCl + cPTIO treatment. The bars represent the means of three independent biological replicates, and standard errors are shown. Statistical significance [least significant difference (LSD)] of the difference in fluorescence intensities is indicated by different small letters (*P* < 0.05).

The NO fluorescence in the root tips of *H. erythrostictum* indicated that 200 mM NaCl caused an abundant NO accumulation (Fig. 6A), and the average fluorescence intensity was about 6.68-fold of that of the control (Fig. 6B). Addition of exogenous NO donor SNP increased the fluorescence brightness in NaCl-treated root tips, and its average fluorescence increased by 68.50%. Moreover, NaCl-induced NO accumulation in the root tips was significantly repressed by the NO scavenger cPTIO and by the NR inhibitor sodium tungstate with about 31.50% and 40.08%, respectively.

**Figure 7:**
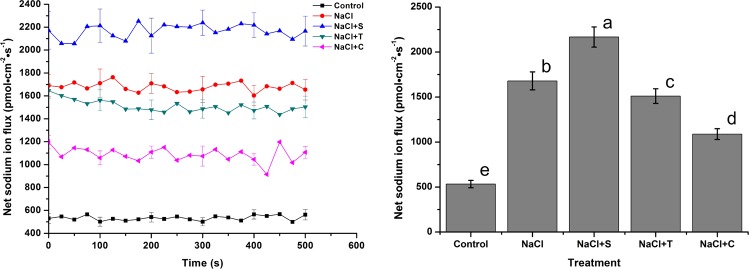
Effect of NaCl and NO reagents on the net Na^+^ flux rate in the root tips of *H. erythrostictum*. (**A**), temporal pattern of ion flux; (**B**), average net ion flux. Positive net ion flux rates indicate ion efflux from cells, while negative net ion flux rates indicate ion influx from cells. Each bar represents the average of five independent replications, and standard errors are shown. Statistical significance [least significant difference (LSD)] of the difference in fluorescence intensities is indicated by different small letters (*P* < 0.05).

The effect of NaCl and NO reagents on the net Na^2+^ flux rate in the root tips was examined by Non-invasive Micro-test Technology (NMT, Fig. 7). After exposure to NaCl, the Na^+^ efflux rate was enhanced. After treatment with NaCl for 24 h, the Na^+^ efflux rate was about 3.15-fold higher than that of the control. The addition of SNP caused a significant 29.06% increase in the net Na^+^ efflux rate compared with NaCl treatment. In contrast, after addition of T and C, the net Na^+^ efflux rates were significantly inhibited. After exposure to NaCl + C, the net Na^+^ efflux rate was 35.21% lower than in NaCl treatment, which was still 2.04-fold higher than that of control.

**Figure 8:**
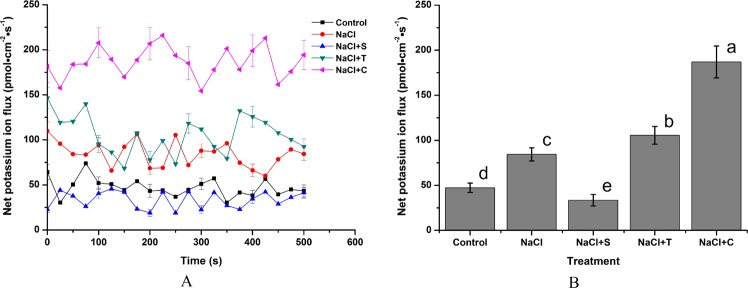
Effect of NaCl and NO reagents on the net K^+^ flux rate in the root tips of *H. erythrostictum*. A, temporal pattern of ion flux; B, average net ion flux.

Figure 8 shows the temporal pattern and average value of the net K^+^ flux rate after exposure to NaCl and NO reagents treatment. A 78.72% increase occurred after a single NaCl treatment. In contrast to Na^+^, NaCl + S treatment significantly decreased the K^+^ efflux rate, which was only 39.29% of the value under NaCl treatment. Treatments of NaCl + T and NaCl + C significantly promoted the K^+^ flux efflux rate compared with NaCl treatment, especially addition of C, which resulted in a 1.22-fold increase.

**Figure 9:**
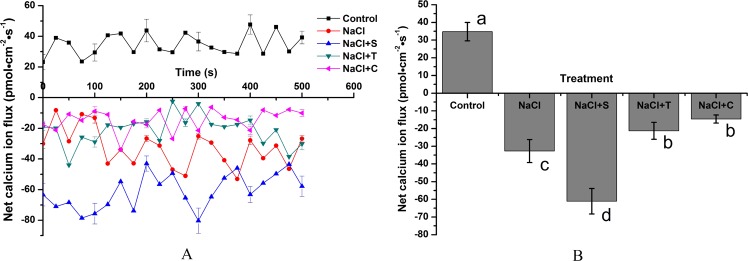
Effect of NaCl and NO reagents on the net K^+^ flux rate in the root tips of *H. erythrostictum*. (**A**), temporal pattern of ion flux; (**B**), average net ion flux.

The effect of NaCl and NO reagents on the net Ca^2+^ flux rate in root tips was also investigated (Fig. 9). In comparison to control, NaCl caused an increase in the net Ca^2+^ influx, which was strongly enhanced by SNP. After treatment with T and L, the increase in the net Ca^2+^ influx rate induced by NaCl was also greatly suppressed. Compared with single NaCl treatment, NaCl + S caused an 86.76% increase in the average net Ca^2+^ influx rate. The average net Ca^2+^ influx rates after treatment with NaCl + T and NaCl + C decreased by 44.98% and 55.29%, respectively, compared to that by single NaCl treatment (*P < *0.05).

## Discussion

Soil salinity is a severe environmental stress factor that strongly reduces plant growth and survival, and thus limits the productivity and quality of agriculture and forestry worldwide. About 20% of irrigated lands have been damaged by high soil salinity, and 1.5 million ha of production reduction each year can be ascribed to soil salinity. Understanding the mechanisms of plant salt resistance, and working toward more salt-tolerant crops for the agricultural and forestry industry is and will be a major research focus in the future. This study uncovered possible salt-resistance mechanisms using RNA-seq to investigate the molecular response to salt stress in the roots of *H. erythrostictum*. Ion transports, including the anion and cation transporters on the plasma membrane of root cells, play a crucial role for the determination of salinity resistance in plants that are exposed to salt stress^31,32^. Under typical physiological conditions, the homeostatic concentrations of ions in the cytosol are 100 to 200 mM K^+^, 1 to 10 mM Na^+^ and C1^−^, and 100 to 200 mM Ca^2+^. Maintaining the balance of intracellular K^+^/Na^+^ is fundamental to the physiology of living cells and is important for normal plant growth^33^. Salt stress increases the Na^+^ level in the plant cells, while a decrease in K^+^ concentration occurs. This disturbs the balance of intracellular K^+^/Na^+^ and leads to osmotic stress and ion toxicity in plants^34^. A primary response in the maintenance of cellular ion homeostasis is the restriction of Na^+^ accumulation and depression of K^+^ depletion in the cytosol. This is done by Na^+^ ion transporters on the plasma membrane and tonoplast^35^. This study identified several unigenes that were annotated to Na^+^ and K^+^ transporters in *H. erythrostictum* roots, including *SOS1, AKT1*, *NHX1*, and *NHX2*, the expressions of which were enhanced in response to salt stress. Two major proteins, that maintain low cytoplasmic Na^+^ concentration in plant cells, are plasma membrane-localized SOS1 and the tonoplast-localized NHX1^36^. *SOS1* encodes a plasma membrane Na^+^/H^+^ antiporter, which is central for Na^+^ efflux from cell^37^, overexpression of which improves the salt tolerance of *Arabidopsis*^38^. The activated expression of *SOS1* in *H. erythrostictum* roots indicated that the SOS pathway was stimulated to export Na^+^ out of the cell. NHX1 controls the movement across the tonoplanst membrane into the vacuole^2^. Overexpressing *NHX1* in salt-tolerant transgenic *Arabidopsis* leads to the strong capacity of Na^+^ sequestration into vacuoles^39^. AtNHX1 was also found to contribute to salt tolerance in *Solanum lycopersicum*^40^ and *Brassica* plants^41^ through increased vacuolar sodium accumulation. AKT1, a major factor for the transport of K^+^ from the external environment by the root epidermis, exhibits the high K^+^/Na^+^ selectivity^42^. AKT1 plays a key role in K^+^ uptake and in the maintenance and restoration of K homeostasis in salt-stressed plants^43^. The transcriptional expression of *AKT1* is significantly up-regulated in roots of NaCl-treated *K. obovata*^44^. Overexpression of *AKT1* is also found to significantly enhance the resistance to high salinity in transgenic *Arabidopsis*^45^. As a high-affinity K^+^ transporter, HKT1 is reported to play a key role in the protect against Na^+^ toxicity through the promotion of K^+^ accumulation^46^. The *Arabidopsis HKT1* expression enhances the activities of SOD, CAT, and POD, increases chlorophyll and soluble sugar contents as well as root activity, decreases MDA and proline contents, reduces electrolyte leakage destruction, and helps to maintain a healthy K^+^ status while reducing Na^+^ toxicity in transgenic tobacco under salt stress^47^. Moreover, *HKT1* also plays a vital role in the plant salt tolerance by controlling the distribution of Na^+^ in the roots and shoots^48^. However, the present study showed that *HKT1* was down-regulated in *H. erythrostictum* roots after exposure to salt stress, which was also the case for *A. officinalis* root *HKT1*^15^. Therefore, in *H. erythrostictum* roots, the enhanced expression of Na^+^ and K^+^ transporters indicated that Na^+^ efflux and K^+^ uptake in the cytoplast were strengthened to maintain the K^+^/Na^+^ balance under salt stress. Moreover, these transporters may cooperate in a whole mechanism to effectively remove Na^+^ from the cytoplast to maintain the K^+^/Na^+^ balance^34,49^. The significant increase in Na^+^ efflux rate in *H. erythrostictum* roots after NaCl treatment demonstrated that Na^+^ transporters extracted excessive Na^+^ out of the cells to decrease the cytosolic Na^+^ concentration. In the roots of *K. obovata* treated with NaCl, a significant enhancement in net Na^+^ efflux is detected^44^. However, after treatment with NaCl, the K^+^ flux in the root tips exhibited an obvious efflux, which was also found in *K. obovata* roots^44^. It has been suggested that salt stress stimulates strong depolarization in plasma membrane, and leads to an influx of excessive Na^+^ to the cytoplasm, and simultaneously increases K^+^ efflux via depolarization-activated channels. Through activation of K^+^ transporters, AKT1 and HKT1 can partly offset K^+^ efflux, and the net K^+^ flux was outward in *H. erythrostictum* roots.

The Ca^2+^ signal plays an important role in plant responses to salt stress^50^. The present study showed that the unigenes annotated to the calcium channel were also highly expressed in *H. erythrostictum* roots under salt stress, and a significant increase in Ca^2+^ influx rate occurred. In NaCl-treated *Bruguiera gymnorrhiza* roots, the Ca^2+^ influx rate is also markedly enhanced^51^. It has been reported that the activated Ca^2+^ channel can cause a rapid increase in cytosolic Ca^2+^ levels within seconds after exposure to NaCl^52^. Mostly, Ca^2+^ accumulation in the root cells functions as the first checkpoint under salt stress^53^. After exposure to NaCl, several genes related to Ca^2+^ signaling are up-regulated in *A. officinalis* roots, thus leading to Ca^2+^ fluxes across plasma membrane and tonoplast^15^. Furthermore, the gene expression levels of calcium binding proteins including CaM, CML, CBL, CIPK, and CDPK were also enhanced by NaCl. The results of the present study indicated that the Ca^2+^ signaling pathway was stimulated to induce resistance responses including protein activation and gene transcription. The expression of *CDPK* of *A. hypogeae* is strongly induced by both NaCl and exogenous Ca^2+^, the overexpression of which enhances the salt-tolerant activity of tobacco^54,55^. In the roots of *A. officinalis*, CaM, CBL, and CIPK are also up-regulated in response to salt stress^15^. Likewise, in *O. sativa*, CML expression is reported to enhance salt tolerance in *Arabidopsis*, which is accompanied by an altered expression of stress/ABA-responsive genes^56^. In addition, the Ca^2+^ signal correlates with the SOS pathway and Na^+^ transporter. CBL interacts with CIPK and builds a complex, which can activate SOS1 and NHX1, thus resulting in Na^+^ efflux across plasma membrane^57^ and Na^+^ compartmentalization into the vacuole^58^, respectively. In the supplementary experiment of this study, the effects of CaCl_2_ and LaCl_3_ on the net fluxes of Na^+^ and K^+^ were measured in *H. erythrostictum* roots (Supplementary Fig. S5). Addition of CaCl_2_ and LaCl_3_ significantly promoted and inhibited the Na^+^ efflux, respectively. In contrast, net K^+^ efflux after NaCl treatment was significantly suppressed by CaCl_2_ and enhanced by LaCl_3_. Thus, the Ca^2+^ signal can regulate the K^+^/Na^+^ balance under salt stress in *H. erythrostictum* roots, and may locate upstream. Overall, these results suggested that Ca^2+^ signaling may play a key role in the salt resistance of *H. erythrostictum*.

NO, as a central signaling molecule, is assumed to play a central role in the plant resistance response to salinity^59^. It has been shown that exposure to exogenous SNP can lead to a better performance in response to NaCl treatment in *O. sativa*^60^, *Lupinus luteus*^61^, *Zea mays*^62^, *Phaseolus vulgaris*^63^, and *K. obovata*^44^. Two main enzyme-based pathways have been proposed to be functional for NO biosynthesis in plants. One pathway is based on the activity of nitrate reductases^64,65^, and the other pathways, still undefined, is based on the direct or indirect function of the Nitric Oxide-Associated1/Resistant to Inhibition by Fosfidomycin1 (AtNOA1/RIF1) protein. By investigating an *Arabidopsis* mutant, AtNOA1 has been proposed to participate in NO biosynthesis^66^. A recent report, characterizing the *Nicotiana benthamiana* homolog of the AtNOA1 gene, fully agrees with this data, since transgenic tobacco plants with VIGS-mediated silencing of NbNOA1 contained reduced levels of NO^67^. In the present study, after NaCl treatment, the expression levels of unigenes annotated to *NOA1* and *NR* increased with time. Moreover, the fluorescence results confirmed NO accumulation after NaCl treatment, which could be further increased by exogenous NO donors and decreased by NO scavengers and NR inhibitors. In the following experiment, to clarify the role of NO in the ion transportation in *H. erythrostictum* roots under salt stress, the effect of NO reagents on the ion fluxes in roots was measured. Under salt stress, SNP significantly increased the Na^+^ efflux rate and decreased the K^+^ efflux rate. When NR were inhibited and NO was scavenged by cPTIO, the efflux rate of Na^+^ decreased, while the efflux rate of K^+^ increased, which meant that the K^+^/Na^+^ ratio decreased. The optimum K^+^/Na^+^ ratio is not only necessary for the activities of many cytosolic enzymes, but also for the maintenance of the optimal membrane potential and osmoticum for cell vital activity^34^. The present study results indicated that NO can modulate the K^+^/Na^+^ ratio to maintain a relatively high level in *H. erythrostictum* roots under NaCl stress, which can enhance the plant’s salt tolerance. Under salt treatment, a previous study has reported that SNP increases Na^+^ extrusion and inhibits K^+^ loss to the external environment at the root apex of *K. candel*^51^. Moreover, NO contributes to the K^+^/Na^+^ balance in NaCl treated *K. obovata* roots by regulating the transmembrane fluxes of K^+^ and Na^+^^43^. NO is also found to mediate the maintenance of K^+^/Na^+^ homeostasis in *G. uralensis* roots during NaCl stress^14^.

In addition, NO has been reported to play a potential role as an endogenous regulator of Ca^2+^ mobilization in plant physiological contexts, which is reported to contribute to the increase in cytosolic Ca^2+^ concentration in plant cells after exposure to biotic and abiotic stresses^68^. In the present study, Ca^2+^ influx induced by NaCl was found to be promoted by SNP and significantly reduced by sodium tungstate and cPTIO in *H. erythrostictum* roots. These results indicate that NO can also act as an upstream signaling messenger to modulate the salt resistance of *H. erythrostictum* through the calcium signaling pathway. Previous pharmacologically-based experiments suggest that NO can activate a transient and fast increase in cytosolic Ca^2+^ concentration by stimulating Ca^2+^-permeable channels on the plasma membrane and tonoplast^69^. Several studies also find that elicitor-induced NO synthesis is controlled by upstream Ca^2+^ influx^68,69^, suggesting an interplay between NO and Ca^2+^. The relationship between NO and Ca^2+^, and how NO regulated the Ca^2+^ fluxes to control the cytosolic Ca^2+^ concentration will be investigated in the following study.

## Conclusions

**Figure 10:**
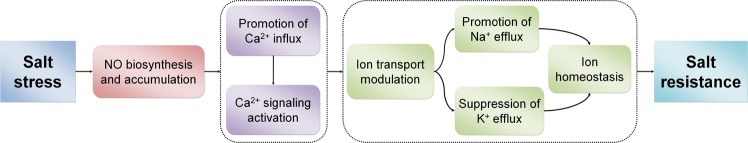
NO regulatory pathway in response to salt stress in *H. erythrostictum* roots.

This study provides the first evidence that in *H. erythrostictum* roots under NaCl treatment, a modulation of cytosolic K^+^/Na^+^ balance by activated K^+^ and Na^+^ transporters occurs as response. The increased gene expression levels of Ca^2+^ channels and Ca^2+^ binding proteins, as well as Ca^2+^ influx acceleration showed that the calcium signaling pathway was also stimulated in response to salt stress, which may play a key role in the K^+^/Na^+^ balance by regulating both K^+^ and Na^+^ transportation. Moreover, NaCl-induced NO may function as a signaling messenger upstream to modulate the K^+^/Na^+^ balance in the cytoplasm via the calcium signaling pathway to enhance the salt resistance of *H. erythrostictum* (Fig. 10).

## Materials and Methods

### Plant materials

This study used the hydroponic seedlings of *H. erythrostictum* as plant materials. The seedlings were cultured in 1/4 Hoagland nutrient solution in an artificial climate chamber under a 14/10 h light/dark 25/18 °C cycle with 150 µmol·m^−2^·s^−1^ light intensity and 60% relative humidity. The culture solution was replaced every three days. When the seedlings grew about 10 leaves, they were treated with NaCl.

### NaCl treatment

The seedlings were treated with 200 mM NaCl, which was prepared using 1/4 Hoagland nutrient solution. The treatment solution was replaced every three days. Root samples were collected after treatment for 0 (T0, control), 5 (T5), and 10 (T10) days, respectively. After being washed with deionized water, and wiped with absorbent paper, the samples were frozen in liquid nitrogen for RNA sequencing and determination of gene expression. Each treatment used three biological replicates.

### Treatment with NO reagents

In this study, 50 μM sodium nitroprusside (SNP)^70^ an exogenous NO donor, 100 μM cPTIO (C)^71^, a NO scavenger, and 100 μM sodium tungstate (T)^72^, a nitrate reductase inhibitor, were used to investigate the role of NO in the response of *H. erythrostictum* root to elevated NaCl levels. NO reagents were added to 200 mM NaCl solution to reach treatment concentrations. Each treatment used three biological replicates.

### Treatment with Ca^2+^ reagents

In this study, 10 mM CaCl_2_ (exogenous Ca^2+^) and 5 mM LaCl_3_ (plasma membrane calcium channel blocker) were used to treat *H. erythrostictum* roots. The Ca^2+^ reagents were added to 200 mM NaCl solution to reach treatment concentrations.

### RNA isolation

Total RNA was isolated using the Trizol method according to the manufacturer’s instructions (Invitrogen). DNase I was added to eliminate DNA to avoid contamination by genomic DNA in the samples. Using Nanodrop-2000, RNA purity was assessed. For each sample, the A260:A280 ratio exceeded 1.8, and the A260:A230 ratio exceeded 2.2.

### Library preparation and Illumina sequencing

Library preparation and high throughput sequencing were performed at Beijing Ori-Gene Science and Technology Ltd. Total RNA were prepared to isolate mRNA. Using Sera-mag Oligo (dT) beads (Thermo), 10 μg of total Poly(A) mRNA was isolated, which was cut into short fragments to establish a paired-end RNA-seq library for transcriptome sequencing. Adapters were added for size selection and PCR amplification. Fragment shortening was interrupted by addition of a stop buffer. using these fragments as templates, the first-strand cDNA was synthesized with a random hexamer-primer. 20 μL second-strand buffer (Invitrogen), 10 mM dNTP Mix, 5 U/μL RNase H, and 10 U/μL DNA polymerase I were used to produce the second-strand cDNA. Using the QIAquick PCR purification kit short fragments were purified, and then resolved in EB buffer for end reparation and poly(A) addition, which was followed by connection with sequencing adapters. Thereafter, suitable fragments were used as templates to be amplified by PCR. Finally, according to Illumina’s protocols, the paired end RNA-seq libraries of the samples were prepared. Then, the constructed libraries were sequenced by the Illumina HiSeq. 2500 platform.

### RNA-seq result quality pre-process and analysis

The data were measured against the Q20 standard and acceptable RNA-seq sequencing quality was obtained. Due to the negative effect on bioinformation analysis, dirty raw reads were removed. Then, the transcriptome was *de novo* assembled, and unigenes were obtained. BLASTX and annotation were performed against six protein databases, i.e., Swiss-Port, TrEMBL, Nr, Pfam, KOG, GO, and KO, with a cut-off E-value of 10^−5^. Based on the KEGG database, the biological complex behaviors of genes were studied, and pathways were obtained that were mediated by unigenes^73,74^. Then, the DEGs were selected according to the fold-changes in gene expression levels with a *p*-value < 0.05.

### Quantitative real-time PCR (qRT-PCR) validation

The unigenes were validated by qRT-PCR. Using SYBR Green PCR Master Mix, the PCR products were detected on a 7900 HT Sequence Detection System (Applied Biosystems), and according to the manufacturer’s protocol, qRT-PCR was performed using the SYBR Premix Ex Taq Kit (TaKaRa). the constitutive actin gene was used as control to normalize to the expression levels of unigenes by a comparative Ct method (2^–△△ct^). Each sample was the same for Illumina sequencing, and used three biological and technical replicates.

### Fluorescence determination of NO

Endogenous NO formation in root tips was visualized using a DAF-FM DA (Sigma-Aldrich) NO-sensitive fluorescent probe and fluorescence stereo microscope. The root tips of control, NaCl treated, and NO reagent treated seedlings were stained with 20 μM DAF-FM DA dissolved in DMSO for 120 min at 4 °C in the dark. Thereafter, root tips were thoroughly washed, and were placed in the dark at 25 °C for 120 min, then, successively analyzed using a fluorescence stereo microscope. The root tips were excited at 488 nm, and emission signals between 505 and 530 nm were collected. Images were processed and analyzed. The green epifluorescence could be attributed to NO production. All images shown represent typical results from at least five replications, and the average fluorescence intensities were calculated. The blank (without DAF-FM DA) and treatment control (without NaCl) were both carried out.

### Measurements of net ion flux rates

After 200 mM NaCl treatment for 5 days, the net flux rates of Na^+^, K^+^, and Ca^2+^ at root tips of *H. erythrostictum* were NMT (NMT100 Series, Younger USA LLC, Amherst, MA, USA; Xuyue Sci. & Tech. Co., Ltd., Beijing, China). The root segments were bathed in test solution for 30 min before measurement to equilibrate the ion environment. Then, the root segments were placed in the measurement chamber containing 10 mL test solution. Since there were differences at different zones, the net ion flux rates at meristematic zone, elongation zone, and maturation zone were determined, and the average values of these three zones were used as results of the root tips. The ion concentration gradient was measured by moving the microsensor between two positions close to the roots in a preset excursion (30 µm) at a programmable frequency in the range of 0.3 Hz. The microsensor was typically corrected three times by calibrations during the experiments. A 10-min continuous recording was performed at each measuring point. Five single-plant replications were conducted for each treatment.

According to the method of Sun *et al*.^75^, the measurement solutions were prepared as follows: the Na^+^ measurement solution contained 0.1 mM NaCl, 0.1 mM MgCl_2_, 0.1 mM CaCl_2_, and 0.5 mM KCl at pH 6.0 (adjusted with choline and HCl); the K^+^ measurement solution contained 0.1 mM NaCl, 0.1 mM MgCl_2_, 0.1 mM CaCl_2_, and 0.5 mM KCl at pH 6.0 (adjusted with choline and HCl); the Ca^2+^ measurement solution contained 0.1 mM NaCl, 0.1 mM MgCl_2_, 0.2 mM CaCl_2_, and 0.5 mM KCl at pH 6.0 (adjusted with choline and HCl). The ion flux rate was calculated using Fick’s law of diffusion:

$$J=-D(dc/dx)$$

Where *J* represents the ion flux rate in the *x* direction, *dc* represents the ion concentration difference, *dx* represents the microelectrode movement between two positions (30 µm), *dc*/*dx* represents the ion concentration gradient, and *D* represents the ion diffusion coefficient in a particular medium. The data were processed by imFluxes V2.0 (Younger USA LLC, Amherst, MA, USA) software. Significant difference among samples was determined using LSD test at *P < *0.05.

## Supplementary information

Supplementary Information
