# Supplementary material for: Nitric oxide modulating ion balance in Hylotelephium erythrostictum roots subjected to NaCl stress based on the analysis of transcriptome, fluorescence, and ion fluxes
Source: Sci Rep. 2019 Dec 4;9:18317. doi: 10.1038/s41598-019-54611-2 (PMC6892800; doi:10.1038/s41598-019-54611-2)
Supplement: Supplementary file 1 — Supplementary Information [file 41598_2019_54611_MOESM1_ESM.pdf]

# Nitric oxide modulating ion balance in *Hylotelephium erythrostictum* roots subjected to NaCl stress based on the analysis of transcriptome, fluorescence, and ion fluxes

Zhixin Chen[1,2], Xueqi Zhao[1,2], Zenghui Hu[1,2,3,4] & Pingsheng Leng[1,2,3]

[1]Beijing Advanced Innovation Center for Tree Breeding by Molecular Design, Beijing University of Agriculture, Beijing, 102206, China
[2]College of Landscape Architecture, Beijing University of Agriculture, Beijing, 102206, China
[3]Beijing Collaborative Innovation Center for Eco-environmental Improvement with Forestry and Fruit Trees, Beijing, 102206, China
[4]Beijing Laboratory of Urban and Rural Ecological Environment, Beijing, 102206, China
Corresponding author: Zenghui Hu, buahuzenghui@163.com, Pingsheng Leng, email: bualengpingsheng@163.com.

**Supplementary Table S1**

**Summary of sequencing and assembly data**: A total of 89.413 Gb of raw base and 596.092 Mb of raw read was obtained from the 9 samples, averaging at 9.935 Gb and 66.232 Mb, respectively.

| Sample | Raw base(G) | Raw read(M) | Clean base(G) | Clean read(M) | Q20(%) | Q30(%) |
|---|---|---|---|---|---|---|
| T0-1 | 9.781 | 65.203 | 8.899 | 62.335 | 99.0 | 96.9 |
| T0-2 | 10.327 | 68.844 | 9.386 | 65.599 | 98.9 | 96.6 |
| T0-3 | 11.908 | 79.388 | 10.8 | 75.688 | 99.0 | 96.8 |
| T5-1 | 8.242 | 54.949 | 7.411 | 52.466 | 99.0 | 96.9 |
| T5-2 | 10.038 | 66.923 | 9.086 | 64.437 | 99.0 | 97.0 |
| T5-3 | 10.209 | 68.063 | 9.288 | 65.377 | 99.0 | 96.9 |
| T10-1 | 11.308 | 75.39 | 10.363 | 72.704 | 99.0 | 97.0 |
| T10-2 | 8.418 | 56.117 | 7.754 | 54.021 | 98.9 | 96.7 |
| T10-3 | 9.182 | 61.215 | 8.536 | 59.092 | 99.0 | 96.8 |
| Average | 9.935 | 66.232 | 9.058 | 63.524 | 99.0 | 96.8 |
| Total | 89.413 | 596.092 | 81.523 | 571.719 | -- | -- |

Note: T0, T5, and T10 indicates that the samples are treated by 200 mM NaCl for 0, 5, and 10 days.

**Supplementary Table S2**

**Length distribution of assembled transcript and unigene**: Discarding low-quality reads, containing adapters and unknown or low-quality bases, and after stringent quality checks and data cleaning, a total of 81.523 Gb clean base and 571.719 Mb of clean read were obtained. The average Q20 and Q3 percentage reached 99.0% and 96.8%, respectively. Based on the high quality reads, using paired-end joining and gap-filling, 177,053 transcripts and 111,341 unigenes were assembled with different length distribution.

| Length range | Transcript | Unigene |
|---|---|---|
| 200-500 | 108,283 (61.16%) | 78,939 (70.90%) |
| 500-1000 | 42,894 (24.23%) | 19,634 (17.63%) |
| 1000+ | 25,876 (14.61%) | 12,768 (11.47%) |
| Total number | 177,053 | 111,341 |
| GC | 46.86% | 47.63% |
| Total Length | 106,463,014 | 59,323,865 |
| N50 Length | 766 | 644 |
| Max Length | 9,725 | 9,725 |
| Mix Length | 224 | 224 |
| Average Length | 601 | 532 |

**Supplementary Table S3**

**Number of enriched GO term and KEGG pathway**: The up-regulated GO terms and KEGG pathways were more than those down-regulated between T0 and T5, T5 and T10, and T0 and T10. By comparing T0 to T5, 933 up-regulated GO terms and 158 KEGG pathways were found, which were 3- and 13-fold of down-regulated numbers, respectively.

| Sample | Enriched GO term | | Enriched KEGG pathway | |
|---|---|---|---|---|
| | Down | Up | Down | Up |
| T0 vs T5 | 441 | 446 | 34 | 61 |
| T5 vs T10 | 301 | 933 | 12 | 158 |
| T0 vs T10 | 438 | 1,065 | 16 | 141 |

**Supplementary Fig. S1**

**The number of unigenes annotated**: All unigenes were aligned to seven protein databases containing Nr, TrEMBL, Swiss-Prot, Pfam, KOG, GO, and KO using BLAST with an E-value threshold of $10^{-5}$. Of 111,341 unigenes annotated, 82,081 (73.7%) unigenes showed significant BLAST matches in the Nr database. Based on comparison against the TrEMBL database, 81,379 (73.1%) unigenes also had significant matches. In the Swiss-Prot, Pfam, KOG, and GO databases, 55,577 (49.9%), 60,384 (54.2%), 67,061 (60.2%), and 68,899 (61.9%) unigenes also have significant matches respectively, and 34,570 (31.0%) unigenes were found to be similar to the proteins in the KO database.

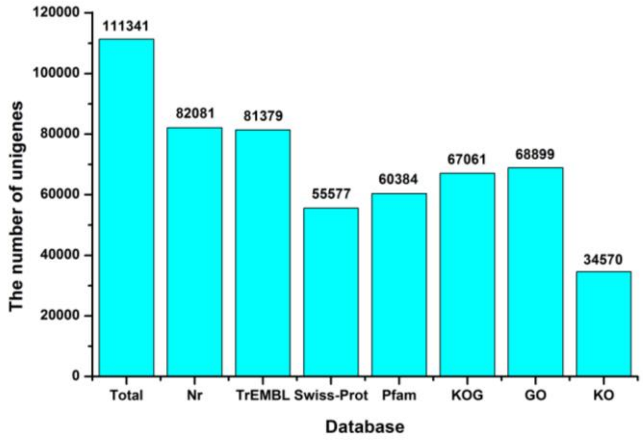

**Supplementary Fig. S2**

**Gene Ontology (GO) classifications histogram of unigenes**: Based on the homology, 68,899 unigenes were categorized into 56 groups. The unigene functions covered the categories of component, molecular function, and biological process. The categories which the unigenes were main assigned were cellular component, biological process, and molecular function. In the category of biological process, the cellular process (43,603, 63.29%) and metabolic process (36,916, 53.58%) represented the majority. A total of 17,299 (25.11%) and 17,131 (24.86%) unigenes were assigned to response to stimulus and biological regulation, respectively. In the category of cellular component, cell (50,206, 72.87%), cell part (50,073, 72.68%), and organelle (37,849, 54.93%) were prominently assinged. In the category of molecular function, binding (40,753, 59.15%) and catalytic activity (34,143, 49.56%) represented the majority. 4,560 (6.62%) unigenes were related to transporter activity. In addition, 484 (0.70%) unigenes were assigned to transcription factor activity, protein binding.

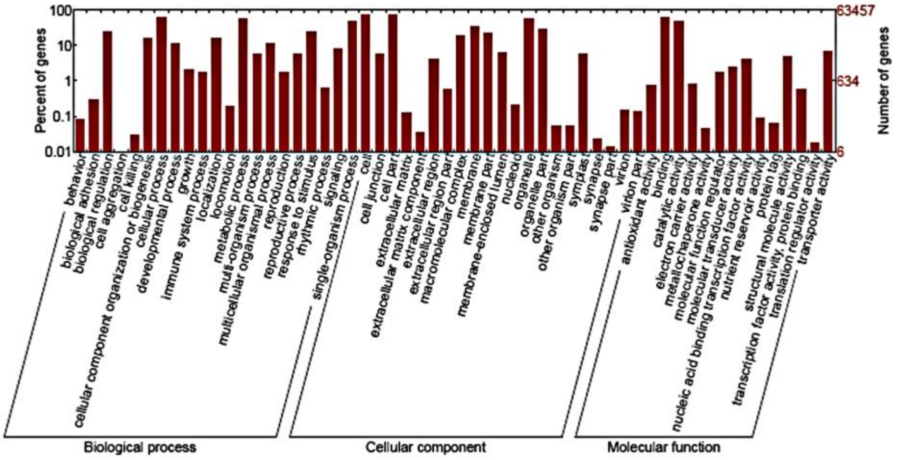

**Supplementary Fig. S3**

**KEGG classification of unigenes**: KEGG database presents the pathways of molecular interactions and reactions, and based on the comparison 34,570 unigenes were assigned to different pathways. Six categories, including Organismal Systems, Metabolism, Human Diseases, Genetic Information Processing, Environmental Information Processing, and Cellular Processes were classified. The percentage of unigenes involved in signal transduction exceeded 10.00%, followed by the unigenes contributing to infectious diseases, and carbohydrate metabolism.

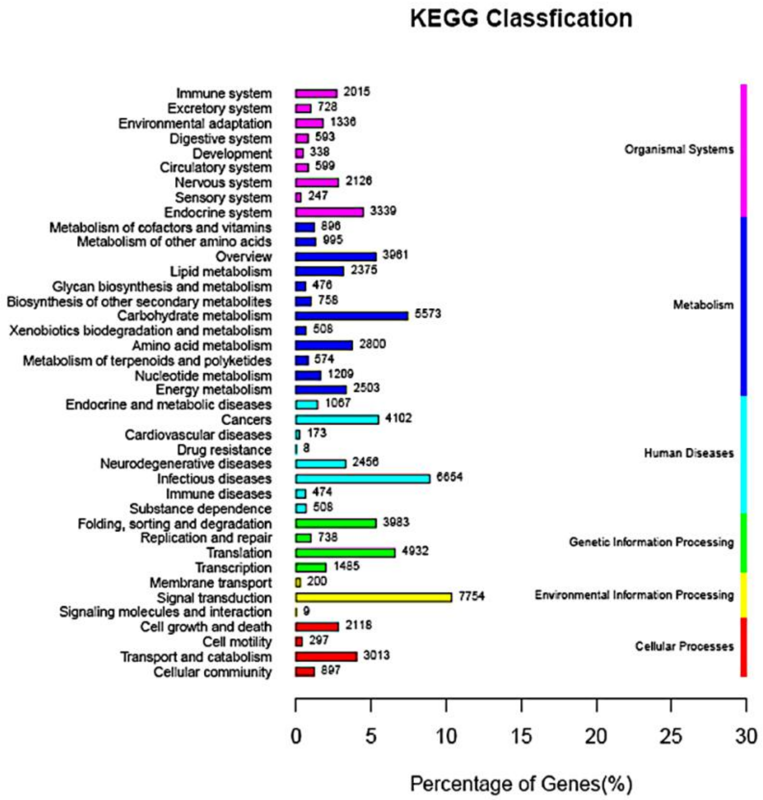

**Supplementary Fig. S4**

**The volcano plot (A) and number (B) of DEGs**: (A) The volcano plots of DEGs between samples were shown. (B) The DEGs were identified between two samples by comparing T0 and T5, T5 and T10, and T0 and T10. 8,517 DEGs between T0 and T5, including 3,633 up-regulated unigenes and 4,884 down-regulated unigenes (T0 vs T5). By comparing T10 to T5, 29,097 DEGs were identified, including 26,276 up-regulated unigenes which almost was 10-fold of down-regulated unigenes (2,821). 31,935 DEGs were found between T0 and T10, including 25,813 up-regulated and 6,122 down-regulated unigenes (T0 vs T10).

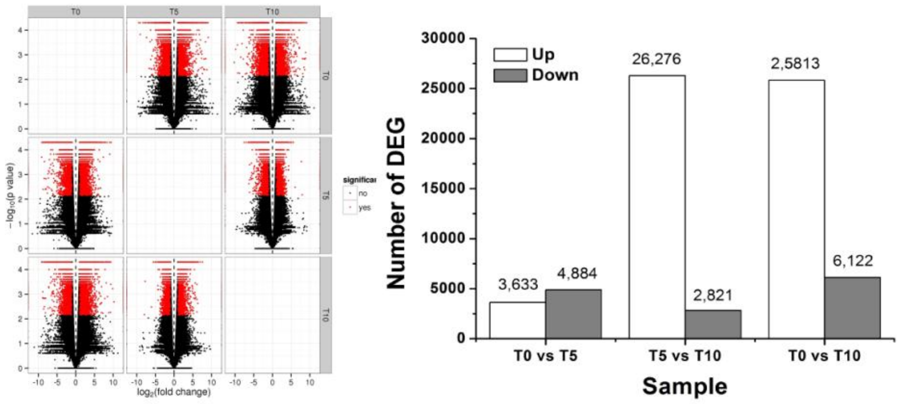


A B

Differentially expressed genes were selected by p-value ≤ 0.05 and |log2 (fold change)|> 1. The x-axis shows the fold change in gene expression between samples, and the y-axis shows the statistical significance of the differences. Splashes represent different genes. Black splashes means genes without significant different expression. Red splashes means significantly differentially expressed genes.

**Supplementary Fig. S5**

**Effect of $Ca^{2+}$ reagents on the net $Na^+$ (A) and $K^+$ (B) fluxes in the root tips of *H. erythrostictum* under NaCl stress:** (A) Addition of $CaCl_2$ and $LaCl_3$ significantly promoted and inhibited the $Na^+$ efflux, respectively. (B) The net $K^+$ efflux after NaCl treatment was significantly suppressed by $CaCl_2$ and enhanced by $LaCl_3$. Thus, the $Ca^{2+}$ signal can regulate the $K^+/Na^+$ balance under salt stress in *H. erythrostictum* roots, and may locate upstream.

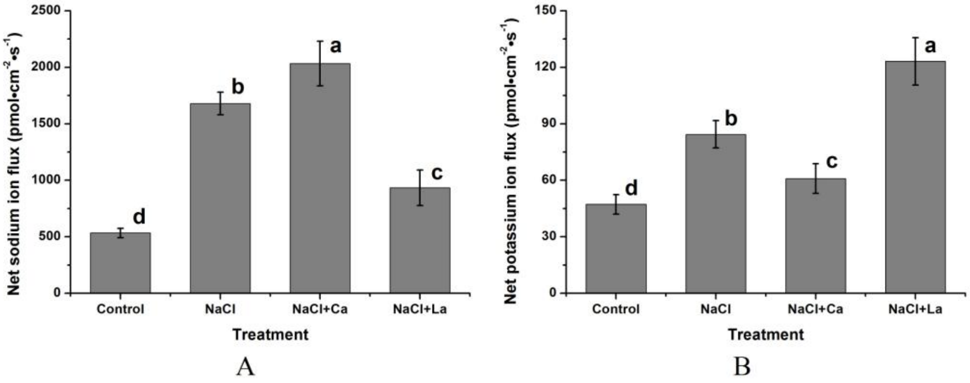


NaCl+Ca, treatment with NaCl + $CaCl_2$; NaCl+La, treatment with NaCl + $LaCl_3$.
